# Characterization of *Schistosoma japonicum* CP1412 protein as a novel member of the ribonuclease T2 molecule family with immune regulatory function

**DOI:** 10.1186/s13071-016-1962-y

**Published:** 2017-02-17

**Authors:** Xue-Dan Ke, Shuang Shen, Li-Jun Song, Chuan-Xin Yu, Mihoko Kikuchi, Kenji Hirayama, Hong Gao, Jie Wang, Xuren Yin, Yuan Yao, Qian Liu, Wei Zhou

**Affiliations:** 1 Key Laboratory on Technology for Parasitic Disease Prevention and Control, Ministry of Health; Jiangsu Provincial Key Laboratory on Technology for Parasite and Vector Control, Jiangsu Institute of Parasitic Diseases, Wuxi, 214064 People’s Republic of China; 2 Department of Immunogenetics, Institute of Tropical Medicine (NEKKEN), Nagasaki University, 1-12-4 Sakamoto, Nagasaki, 852-8523 Japan; 3 Medical College, Jiangnan University, Wuxi, 214122 China; 4 Public Health Research Center, Jiangnan University, Wuxi, 214122 People’s Republic of China; 5 Shanghai Jiao Tong University Affiliated Sixth People’s Hospital, Shanghai, 200233 People’s Republic of China; 6 Department of Pathology, Nanjing Drum Tower Hospital, Nanjing, 210003 People’s Republic of China

**Keywords:** *Schistosoma japonicum*, *Sj* CP1412, RNase activity, Th2 polarizaton, CD4 + CD25 + Foxp3 + T cell

## Abstract

**Background:**

Schistosome infection typically induces a polarized Th2 type host immune response. As egg antigen molecules play key roles in this immunoregulatory process, clarifying their functions in schistosomiasis would facilitate the development of vaccine and immunotherapeutic methods. *Schistosoma japonicum* (*Sj*) CP1412 (GenBank: AY57074.1) has been identified as a new member of the RNase T2 family with immune regulatory functions.

**Methods:**

The expression plasmid *Sj* CP1412-pET28a was constructed and transformed into bacteria for production of recombinant *Sj* CP1412 protein (r*Sj* CP1412) *via* IPTG induction. The RNase activity of *Sj* CP1412 was predicted by bioinformatic analysis and confirmed by digesting the yeast tRNA with r*Sj* CP1412.C57BL/6j mice were immunized with r*Sj* CP1412, and its immune regulatory effects in vivo and in vitro were investigated. Meanwhile, the relationship between the RNase activity of *Sj* CP1412 and its immune regulation was observed.

**Results:**

*Sj* CP1412 was confirmed as a novel RNase T2 family protein with RNase activity. Immunoblotting and RT-PCR analyses demonstrated *Sj* CP1412 as a protein exclusively secreted/excreted from eggs, but not cercariae and adult worms. Stimulating RAW264.7 macrophages with r*Sj* CP1412 raised the expression of CD206, Arg-1 and IL-10, which are related to M2 type macrophage differentiation. Stimulating dendritic cells (DCs) with r*Sj*CP1412 failed to induce their maturation, and the recombinant protein also inhibited LPS-stimulated DC maturation. Depletion of *Sj* CP1412 from soluble egg antigen (SEA) impaired the ability of SEA to induce M2 type polarization of RAW264.7 macrophages. Immunizing mice with r*Sj* CP1412 induced high antibody titers, increased serum IL-4 and TGF-β levels and splenic CD4 + CD25 + Foxp3 + T cells, downregulated serum IFN-γ levels and alleviated the egg granuloma pathology of schistosome infection. In vitro stimulation by r*Sj* CP1412 significantly increased CD4 + CD25 + Foxp3 + T cell numbers in splenocytes of healthy mice. The r*Sj* CP1412 protein with RNase activity inactivated by DEPC failed to induce M2 surface marker CD206 expression in RAW264.7 macrophages.

**Conclusions:**

The *Sj* CP1412 protein expressed specifically in *S. japonicum* eggs is a novel member of the RNase T2 family. Similar to Omega-1 of *Schistosoma mansoni*, the *Sj* CP1412 protein drives polarization of the host Th2 immune response, which is dependent on its RNase activity. These data provide new evidence towards understanding the immune regulatory role of RNase T2 family proteins during schistosome infection.

**Electronic supplementary material:**

The online version of this article (doi:10.1186/s13071-016-1962-y) contains supplementary material, which is available to authorized users.

## Background

Schistosomiasis is prevalent in 76 countries and regions of Asia, Africa and South America, with nearly 600 million people worldwide threatened by the risk of schistosome infection. More than 200 million people are affected by schistosomiasis, 12 million of whom have serious clinical symptom. The number of deaths each year due to schistosomiasis amounts to more than 200,000 individuals [1–3]. Therefore, this disease seriously compromises the health of affected individuals and greatly hinders the local social and economic development in endemic areas.

The effort to control schistosomiasis still mainly depends on the application of praziquantel, which is currently the only effective chemotherapeutic drug and has been in widespread use worldwide for more than 30 years. The long-term large-scale repeated use of praziquantel has resulted in schistosomes with low drug sensitivity or resistance [4, 5], which would hinder the prevention and control of schistosomiasis. The successful control of many infectious diseases has depended on the development and reasonable application of an efficient vaccine. Thus, clarifying the immune mechanism and molecular basis of schistosome infection will be helpful for developing effective vaccines and immune therapeutic measures for preventing and controlling schistosomiasis.

The host immune response has been shown to shift from a Th1 type to Th2 type during schistosome infection, and the downregulation of immune function ultimately leads to chronic infection [6–9]. During the first 3–5 weeks of schistosome infection, the host forms a low level Th1 immune response with elevated levels of inflammatory cytokines IFN-γ, IL-12 and TNF-α [10, 11]. Thereafter, the Th1 immune response is gradually suppressed and the Th2 immune response is enhanced as the adult worms become mature and lay eggs, the serum cytokine levels of IL-4, IL-5, IL-13 and TGF-β in the host begin to rise [10, 11]. The Th2 immune response is also downregulated in the chronic period [12].

The schistosome infection downregulates the host immune function, which provides a balanced co-existence between the host and parasites. The alleviation of immune changes during schistosome infection protects the host from inflammatory damage and death, as well as prevents the parasites from being eliminated by the host immune response. However, the molecular mechanism of this complex immune interaction between the host and schistosomes is not fully understood.

Schistosomes have developed a variety of strategies to evade the host immune attack and drive the host immunity towards the Th2 response in the process of infection [13]. These strategies mainly include changing the activation status of dendritic cells (DCs) and macrophages. For example, the activation and maturation of DCs can be inhibited by downregulating expression of surface co-stimulatory molecules to interrupt their antigen presentation function [14–16]. Macrophages can be induced to differentiate toward the M2 type (i.e. alternatively activated macrophages [17–20]) and accumulate in the tissue around eggs, thereby regulating the activation of other immune cells [21] to reduce inflammation. The schistosomes also can upregulate a variety of cytokines related to the Th2 type response, such as IL-4, IL-10, TGF-β and TNF-α [22]. Furthermore, they can induce cell apoptosis to eliminate the CD4 + T cells activated by schistosome antigens [23] and increase the abundance of eosinophilic and CD4 + CD25 + Foxp3 + T regulatory (Treg) cells [24, 25].

Baumgart et al. [25] found that the number of CD4 + CD25 + Foxp3 + Treg cells at the 4th week after infection of mice with *Schistosoma mansoni* was increased significantly. As the infection progressed, the Treg cells expanded further in the mesenteric lymph nodes and gathered in the liver and spleen of mice. Another study demonstrated that injection of mice with live eggs, dead eggs or the soluble egg antigen (SEA) of *S. mansoni* could significantly induce CD4 + CD25 + Foxp3 + T cell activation [6], indicating that certain components of the schistosome egg antigens can induce polarization of the Th2 response and increase Treg cells. Since only the antigens that are released into the host tissue and circulating blood can possibly come into contact with the host immune cells to induce an immune response, the secreted/excreted protein molecules from eggs may be the major immune regulatory components. Identifying the excreted egg proteins involved in modulating the host immune response will help to clarify not only the molecular mechanisms underlying the negative regulation of immune function, but also the formation of egg granulomas and liver fibrosis in schistosome infection.

The glycoprotein molecules of SEA have been found to be indispensable key components for inducing the Th2 immune response [26]. Dunne et al. [27] divided the *S. mansoni* SEA into six components by ion exchange chromatography and showed that the sixth component (CEF6) contains Omega-1 and alpha-1 (also known as IPSE). Thereafter, Omega-1 was found to be a strongly immunogenic hepatic toxin component, since the anti-serum to Omega-1 could prevent liver cell damage during schistosome infection of T cell deficient mice [27, 28]. Omega-1 is a member of the ribonuclease T2 family with RNA degradative enzyme activity [29]. The Omega-1 molecule can enter DCs *via* binding of its sugar chain to the mannose receptor (MR) on the surface of DCs and degrade the mRNA of ribosomes to inhibit synthesis of cellular proteins which mediate the DC function of priming the Th2 type immune response [30, 31]. The other highly antigenic glycoprotein alpha 1 (IPSE) of the CEF6 components of *S. mansoni* SEA can combine with IgE to activate the alkaline granulocyte releasing histamine and promote these cells to produce Th2 type cytokines, such as IL-4 and IL-13 [32].

The host immune response to *Schistosoma japonicum* is similar to that to *S. mansoni* infection, typically featuring Th2 polarization after egg laying. The major immune regulatory molecules of Omega-1 and IPSE in *S. mansoni* eggs have not been found in *S. japonicum* eggs. Therefore, the *S. japonicum* egg antigens inducing Th2 polarized responses and related mechanisms are still poorly understood. Our group previously screened a batch of genes encoding secreted proteins with a signal sequence from a *S. japonicum* egg cDNA library by the Signal Sequence Trapping (SST) method [33]. Among them, the *Sj* CP1412 protein was found by amino acid sequence alignment to contain the conserved functional domains (CAS-1 and CAS-2) of the ribonuclease T2 family. Although the homology between amino acid sequences of the *Sm* Omega-1 and *Sj* CP1412 molecules is low, *Sj* CP1412 was hypothesized to be another novel member of the ribonuclease T2 family in schistosomes. In the current study, the recombinant *Sj* CP1412 protein (r*Sj* CP1412) was prepared by a prokaryotic expression system and demonstrated to have ribonuclease activity, which could inhibit DC cell activation, promote M2 macrophage differentiation, increase the number of Treg cells and drive the Th2 polarization of the host immune response. These findings support *Sj* CP1412 as an important immune regulatory factor in *S. japonicum* eggs.

## Methods

### Experimental animals

Female C57BL/6j mice at 6 weeks of age and weighing 20–22 g were purchased from the Experimental Animal Center of Yangzhou University (Yangzhou, China). Japanese white rabbits were provided by the Jinling Animal Center (Nanjing, China). All animals were raised at the Department of Experimental Animals, Jiangsu Institute of Parasitic Diseases (Wuxi, China). Animals were sacrificed by CO_2_ anesthesia in a sealed container according to the guidelines. All efforts were made to minimize discomfort and suffering of animals.

### Parasites and antigens

Schistosome-infected snails (*Oncomelania hupensis*) were provided by the Department of Snail Biology, Jiangsu Institute of Parasitic Diseases, China. *Schistosoma japonicum* cercariae were obtained by immersing schistosome-infected snails in dechlorinated water at 25 °C with illumination for 2–4 h to stimulate cercarial emergence. Each rabbit was infected with 1000 to 1500 larvae through the abdominal skin, and the adult worms were collected at day 45 post-infection by portal vein perfusion with anticoagulant saline (0.3% sodium citrate, 0.7% sodium chloride). The eggs were separated and collected by filtration of the homogenate of the infected rabbit liver tissue in cold 1.2% saline through a 160–200 mesh (equal to 96–75 μm) sieve. The excreted and secretory antigens of eggs (ESA) were prepared by incubating the eggs in a small amount of 1.2% saline at 4 °C for 8 h. After centrifugation at 300× *g* for 15 min, the supernatant containing the ESA was collected. The soluble adult worm antigen (AWA), soluble cercaria antigen (SCA) and SEA were also prepared by homogenizing adult worms, cercariae and eggs in sterile PBS, respectively. After repeated freezing/thawing and homogenization, the homogenates were centrifuged at 12,000× *g* for 30 min at 4 °C. The supernatants of homogenates of adult worms, cercariae and eggs were collected separately. Protease inhibitor (100 U/ml, Sigma-Aldrich, St. Louis, MO, USA) and phenylmethylsulfonyl fluoride (PMSF, 1 mmol/l) were added into the ESA, AWA, SCA and SEA solutions. Protein concentrations of ESA, AWA, SCA and SEA were determined using a BCA kit (Pierce Biotechnology, Rockford, IL, USA) before storing the samples at −80 °C. The *Sj* CP1412 depleted SEA was also prepared by the immune magnetic bead adsorption method using magnetic beads labeled with an IgG antibody against r*Sj* CP1412, which were prepared following the instructions of the manufacturer of the GoldMag Nano gold magnetic particles protein coupling kit (Xian GoldMag Nanobiotech Co., Ltd, Xian, China). The IgG antibody against r*Sj* CP1412 was prepared from the immunized mice serum with *Staphylococcus aureus* A protein affinity chromatography resin (Genescript Biological Technology Co., Ltd., Nanjing, China), The *Sj* CP1412 depleted SEA was checked for residual *Sj* CP1412 by western blotting, and no corresponding protein band was observed (see Additional file 1: Figure S1). The endotoxin of SEA, r*Sj* CP1412 and *Sj* CP1412 depleted SEA was removed with Detoxi-Gel™ Endotoxin Removing Gel of Thermo Scientific following the manufacturer’s instruction respectively, and its level was lower than 0.25 EU/ml.

### Cells and plasmids

The RAW246.7 mouse macrophage cell line was provided by Chuan Su, Department of Pathogenic Biology, Nanjing Medical University, Jiangsu Province, China. The plasmid pET28a (+) was obtained from Novagen (Madison, WI, USA). Host bacteria strains *Escherichia coli* DH5α and *E. coli* BL21 (DE3), as well as the recombinant plasmid pEU-GST-CP1412 containing the *Sj* CP1412 gene, were maintained by the authors.

### Cloning of gene encoding the *Sj* CP1412 mature peptide, construction of recombinant expression plasmids and bioinformatic analysis

Gene-specific primers for amplifying the DNA fragment encoding the mature peptide of *Sj* CP1412 were designed and synthesized according to the published gene sequences of *Sj* CP1412 (AY57074.1). The primer sequences were designed as follows: upstream primer P1 (5'-TAG GAT CCA GTG TTC AGG ACA ATT CA-3') and downstream primer P2 (5'-ATC TCG AGC ACA CTG GTT GAT AAT T-3'), with the restriction enzyme sites *Bam*H I and *Xho* I (Promega, Madison, WI, USA), respectively, introduced into the 5' end of the primers. A DNA fragment of ~666 bp was amplified by conventional PCR from the pEU-GST-CP1412 plasmid as DNA template and cloned into the pET28a (+) plasmid at *Bam*H I and *Xho* I enzyme sites to construct the recombinant expression plasmid pET28a-*Sj* CP1412. The inserted target gene fragment in the recombinant expression plasmid was confirmed by DNA sequence analysis.

The signal sequence of *Sj* CP1412 was predicted by using the online SignalP 4.0 server (http://www.cbs.dtu.dk/services/SignalP/). The NetNGlyc 1.0 server (www.cbs.dtu.dk/services/NetNGlyc/) was used for predicting N-glycosylation sites, while the Compute pI/mW tool (http://web.expasy.org/compute_pi/) was used for the calculation of molecular weight and isoelectric point of *Sj* CP1412. Sequence homology searches for *Sj* CP1412 were conducted through the NCBI BLAST Server. Amino acid identities between *Sj* CP1412 and other members of the ribonuclease T2 family of schistosomes were analyzed by multiple alignments usingthe GeneDoc software (version 2.7.0).

### Expression and purification of recombinant *Sj* CP1412 protein

The recombinant expression plasmid pET28a-*Sj* CP1412 was transformed into the *E. coli* BL21 (DE3) competent cells and then selected by incubation on Luria-Bertani (LB) medium plates containing kanamycin (50 μg/ml) at 37 °C overnight. Single colonies from the LB medium plates were transferred to 3 ml LB medium (containing kanamycin 50 μg/ml) and incubated at 37 °C overnight with shaking. The following day, the bacteria cultures each were subcultivated into 1 l of fresh LB medium containing kanamycin 50 μg/ml by 1:100 dilution and then continued growth at 37 °C with shaking at 200 rpm. When the OD600 nm value of the bacteria reached 0.35–0.5, IPTG was added to the final concentration of 1 mM, and the cultures were further incubated with shaking for 4 h. The bacteria were collected by centrifuging the culture at 5000 rpm for 10 min at 4 °C. The pelleted bacteria were resuspended in 100 ml PBS and subjected to repeated freezing and thawing. Subsequently, the bacteria were ultrasonicated in an ice bath (power, 200 W) six times for 20 s each time with intervals of 20 s. The ultrasonicated lysates were centrifuged at 12,000× *g* for 15 min at 4 °C, and the supernatants and precipitates were collected separately. The r*Sj* CP1412 was found to be expressed as an insoluble inclusion body protein in the precipitated lysates by 12% SDS-PAGE analysis. To prepare the purified r*Sj* CP1412 protein, the precipitate was washed with 100 ml PBS three times to remove the soluble protein, and then denaturation buffer (LE Buffer, Genescript Biological Technology Co., Ltd., Nanjing, China) containing 8 M urea was added to dissolve the precipitate by magnetic stirring. After the precipitate was dissolved completely, the solution was centrifuged at 12,000× *g* for 30 min at 4 °C. The supernatant was collected carefully and then passed through a 0.45-μm filter to remove the residual particles. The effluent was loaded on a Ni-IDA affinity chromatography column to purify the denatured r*Sj* CP1412 protein following the manufacturer’s instruction. The purified r*Sj* CP1412 protein was renatured by gradient dialysis with PBS containing urea at different concentrations (6, 5, 4, 3, 2, 1 and 0 M) at 4 °C overnight. Finally, the renatured soluble r*Sj* CP1412 protein was recovered by centrifuging the dialyzed solution at 12,000× *g* for 30 min at 4 °C to remove the insoluble precipitate. The protein concentration was determined by the BCA^TM^ Protein Assay Kit (Thermo Scientific Pierce, Rockford, IL, USA), and the protein purity was analyzed by SDS-PAGE.

### Preparation of *Sj* CP1412 anti-serum and western blotting

The purified r*Sj* CP1412 was mixed with Freund’s adjuvant to form a water-in-oil adjuvant antigen. Each mouse was inoculated with 100 μl of complete Freund’s adjuvant (Sigma-Aldrich, St. Louis, MO, USA) antigen (including 100 μg r*Sj* CP1412 protein) by intracutaneous immunization in the back of the neck skin for the first innoculaton. Boosters were given two times at 2 week intervals using incomplete Freund’s adjuvant antigen with the same amount of r*Sj* CP1412 protein. Orbital venous blood samples were collected two weeks after the final immunization, and the sera were separated for titering IgG antibodies against r*Sj* CP1412 protein by the conventional enzyme-linked immunosorbent assay (ELISA).

The same amounts of r*Sj* CP1412, AWA, SCA, SEA and ESA samples were separated by 12% SDS-PAGE and then transferred to nitrocellulose (NC) membranes (Sigma-Aldrich, St. Louis, MO, USA). The membranes were then blocked with 5% skim milk in PBS with 0.05% Tween-20 (PBST) for 2 h at room temperature, followed by incubation with the mouse sera against the r*Sj* CP1412 protein (diluted by 1:100 with PBST) at room temperature for 2 h. The NC membranes were washed three times with PBST for 10 min each time and then reacted with the goat anti-mouse HRP-conjugated secondary antibody (Bethyl Laboratories, Montgomery, TX, USA) at 1:3000 dilution for 2 h at room temperature, followed by three washes as above. The recognized protein bands were visualized with an ECL chemiluminescence kit following the manufacturer’s instructions (BeyoECL Plus, BiYunTian Biotechnology, Nantong City, China). Light signals of recognized protein bands were detected by using a BIO-RAD chemiluminescence imaging system (ChemiDoc™ XRS + System with Image Lab™ Software).

### Preparation of RNA and RT-PCR

Total RNA samples of cercariae, adult worms and eggs of *S. japonicum* were prepared with the Qiagen RNeasy Mini Kit (Qiagen, Valencia, CA, USA) following the manufacturer’s instructions. The first chain cDNAs of cercariae, adult worm and egg were synthesized with the Superscript^TM^ III First Strand Synthesis System (Invitrogen, Carlsbad, CA, USA). The mRNA levels of *Sj* CP1412 protein were detected by PCR using the same amount of first strand cDNA from the adult worms, cercariae and eggs of *S. japonicum* as template and the *Sj* CP1412 gene-specific primers (P1, P2). Ten microliters of each amplification product was analyzed by 1% agarose gel electrophoresis with ethidium bromide.

### Analysis of r*Sj* CP1412 enzymatic activity

Yeast RNA (1 mg) (Invitrogen) was dissolved in 1 ml reaction buffer (50 mmol/l Tris–HCl, 50 mmol/l NaCl, pH 7.0) to prepare a stock solution (1000 ng/l). Different amounts of purified r*Sj* CP1412 protein (1.25, 2.5 and 5 μg) were then mixed with yeast RNA (200 ng) and incubated at 37 °C in a water bath for 1 h. Meanwhile, commercial RNase was used as the positive control, and undigested yeast RNA was the negative control. The yeast RNA sample was also digested with a recombinant surface antigen 1 (SAG1) of *Toxoplasma gondii* (which was prepared by our laboratory, with no predicted RNase activity) by using the same method to remove the possibility of RNase contamination. The RNase activity of r*Sj* CP1412 was observed by analyzing the enzymolysis products by 1% agarose gel electrophoresis with ethidium bromide.

### Stimulation test of r*Sj* CP1412 protein on RAW264.7 macrophages in vitro

RAW264.7 macrophages (1 × 10^6^) were co-cultured with r*Sj* CP1412 (40 μg/ml), SEA (40 μg/ml), *Sj* CP1412 depleted SEA (40 μg/ml) and PBS at 37 °C with 5% CO_2_. After 24 h of incubation, cells and supernatants were collected. Cells were washed with PBS and separated into the following groups and incubated with the appropriate antibody for analysis: (i) no antibody; (ii) FITC-CD16/32 antibody; (iii) FITC-CD206 antibody; (iv) PECy5-F4/80 antibody; (v) PECy5-F4/80 antibody, FITC-CD16/32 antibody; (vi) PECy5-F4/80 antibody, FITC-CD206 antibody (eBioscience, San Diego, CA, USA). At the same time, normal macrophages were used as controls. All cells were incubated at 4 °C for 30 min in the dark and then washed with PBS before detecting the macrophage surface markers using a BD™ FACScalibur flow cytometer (BD Biosciences, San Jose, CA, USA).

The cytokine levels of IL-10 and IL-12 in the culture supernatant of RAW264.7 cells stimulated by the r*Sj* CP1412 protein were measured using corresponding commercial ELISA kits following the manufacturer’s instructions (Beijing 4A Biotech Co., Ltd., Beijing, China). Total RNA of macrophages was extracted by using the Qiagen RNeasy Mini Kit, and then the first chain cDNA was synthesized with the Superscript^TM^ III First strand Synthesis System. The mRNA levels of induced nitric oxide synthase (iNOS) and arginase-1 (Arg-1) of different macrophage groups were detected by PCR with equal amounts of the first chain cDNA template and corresponding gene-specific primers. Meanwhile, the mouse glyceraldehyde-phosphate dehydrogenase (GAPDH) gene was amplified as an internal control. Sequences for the gene-specific primers were as follows: iNOS (forward: 5'-CGG ATG GAT AGG AAC CTG-3', reverse: 5'-GGA GTG CCT GTG TGC ACC TGG AA-3'; product length, 311 bp); Arg-1 (forward: 5'-CAG ATA TGC AGG GAG TCA CC-3', reverse: 5'-CAG ATA TGC AGG GAG TCA CC-3'; product length, 250 bp); GAPDH (forward: 5'-CTG CAC CAC CAA CTG CTT AG-3', reverse: 5'-GTC TGG GAT GGA AAT TGT GA-3'; product length, 660 bp). Ten microliters of each PCR product was analyzed by agarose gel electrophoresis to determine the mRNA expression levels of iNOS and Arg-1 in RAW264.7 macrophages stimulated by the r*Sj* CP1412 protein.

### Analysis of stimulatory effects of r*Sj* CP1412 protein on DCs in vitro

Bone marrow cells of C57BL/6 J mice were collected by aseptic manipulation, and the cell density was adjusted with RPMI1640 medium to 2 × 10^6^/ml. One milliliter of the cell suspension was transferred to a six-well cell culture plate, and then RPMI1640 medium containing rmGM-CSF (10 ng/ml), rmIL-4 (5 ng/ml) (Peprotech, Rocky Hill, NJ, USA) and 10% FBS was added to a total volume of 5 ml. The cells were cultured in a 37 °C incubator with 5% CO_2_, and the cell culture medium was replaced with fresh medium containing the same concentrations of rmGM-CSF and rmIL-4 at 48 h, thereby removing the suspended cells and retaining only adherent cells. At 72 h and 120 h, half of the medium was replaced with fresh culture medium (with the same concentrations of rmGM-CSF and rmIL-4), retaining as many of the suspended cells as possible. The mouse cells which were the enriched bone marrow-derived DCs (BMDCs) were suspended by gently blowing with a pipette and collected at 168 h. The purity of BMDCs was determined by staining with a PE Cy5-anti-CD11c antibody and detected by FACS.

The DC density was adjusted to 1 × 10^6^/ml with complete RPMI1640 medium, and 1 ml of the cell suspension was transferred to each well of a 24-well cell culture plate. Different groups of cells were in vitro stimulated with the following individual or combined antigens: LPS (100 μg/ml), SEA (40 μg/ml), r*Sj* CP1412 (40 μg/ml), *Sj* CP1412 depleted SEA (40 μg/ml), SEA plus LPS, r*Sj* CP1412 plus LPS and *Sj* CP1412 depleted SEA plus LPS. The stimulation was repeated three times. The cells were cultured in a 37 °C incubator with 5% CO_2_ for 48 h. Thereafter, the cells and supernatants were collected, and the cells were washed once with PBS. The cells were divided into three parts and stained with the following antibodies: (i) PE-Cy5 CD11c antibody and FITC-MHCII antibody; (ii) PECy5-CD11c antibody and FITC-CD80 antibody; (iii) FITC-CD11c antibody and PE-CD86 antibody (eBioscience). At the same time, non-stimulated cells were stained by the same methods as the isotype control. All cells were incubated at 4 °C in the dark for 30 min and then washed with PBS. The DC surface markers were detected with a BD™ FACScalibur flow cytometer. IL-10 and IL-12 cytokine levels in the supernatant of DC cell cultures were measured by the corresponding ELISA (Beijing 4A Biotech Co., Ltd.).

### Effect of r*Sj* CP1412 protein on immune responses of mice in vivo

C57BL/6 J mice were grouped randomly, with ten mice in each group, and immunized with r*Sj* CP1412, SEA or PBS. The immunization strategy was the same as above, and five mice of each group were infected with 30 cercariae on day 14 after the third immunization. The orbital venous blood samples of non-infected mice were collected, and the serum was isolated on day 7 after the third immunization. Levels of TGF-β, IL-10, IL-4 and IFN-γ in mouse sera were detected by corresponding cytokine ELISA kits following the manufacturer’s instructions (Beijing 4A Biotech Co., Ltd.). The immunized mice were sacrificed, and the spleens were excised to prepare single cell suspensions by aseptic manipulation. The proportion of CD4 + CD25 + Foxp3 + Treg cells in 2 × 10^6^ spleen cells was determined using a Treg cell detection kit (eBioscience) following the product manual.

Single spleen cell suspensions of healthy mice also were prepared and adjusted to 5 × 10^6^ cells/ml. Each cell suspension was transferred to a 24-well cell culture plate in 1 ml per well. SEA (40 ng/μl), r*Sj* CP1412 (40 ng/μl) or PBS was added to the wells, and each stimulation was repeated three times. The cells were cultured in a 37 °C incubator with 5% CO_2_ for 48 h and then centrifuged at 1000 rpm for 10 min before collecting both cells and culture supernatants. Concentrations of TGF-β, IL-10, IL-4 and IFN-γ in the culture supernatants were measured using corresponding cytokine ELISA kits following the manufacturer’s instructions (Beijing 4A Biotech Co., Ltd.). The change in percentage of CD4 + CD25 + Foxp3 + Treg cells in the stimulated spleen cells was determined using a Treg cell detection kit following the product manual.

To observe the liver tissue pathology after r*Sj* CP1412 immunization, the infected mice above were sacrificed on day 42 after infection. The liver tissues were removed, fixed with formalin and sectioned for hematoxylin and eosin (HE) staining. Egg granulomas containing a whole mature miracidium egg in the center were selected, and 25 single egg granulomas from the animals of each group were measured. The mean areas of egg granulomas of each group were calculated. Single spleen cell suspensions of the infected mice were prepared and stimulated with r*Sj* CP1412 as above, and the spleen cell proliferation rates were measured by using a cell growth determination kit following the manufacturer’s instructions. Meanwhile, concentrations of IFN-γ and IL-4 in the cultured supernatants of the stimulated cells were measured after 48 h.

To observe the effects of r*Sj* CP1412 immunization on the type of immune response during early infection in mice, 30 mice were divided into healthy (no treatment), infection (infected with 30 cercariae) and r*Sj* CP1412 immunization/infection (immunized first with r*Sj* CP1412 as above, then infected with 30 cercariae at 14 days after final immunization) groups. ELISAs were used to determine levels of IFN-γ and IL-4 in mouse serum and in the culture supernatant of splenic cells, which were stimulated with r*Sj* CP1412, SEA or LPS, from healthy, infection and r*Sj* CP1412 immunization/infection groups at day 21 post-schistosome infection. The liver egg granuloma pathology also was observed by tissue sectioning and HE staining.

### Effects of DEPC-inactivated r*Sj* CP1412 on macrophage surface markers

The r*Sj* CP1412 protein was shown to lose its ribonuclease activity by incubation in buffer containing DEPC (see Additional file 2: Figure S2). After using inactivated r*Sj* CP1412 to stimulate RAW264.7 macrophage cells in vitro as above, the CD206 surface marker of macrophages was analyzed by FACS. Meanwhile, non-stimulated cells were used as negative controls, and r*Sj* CP1412 stimulated cells were used as positive controls.

### Statistical analysis

Statistical comparisons were conducted in Prism (GraphPad Software). Student’s *t*-test was used to determine statistical significance when two groups were compared and Chi-square test was used to determine statistical significance for rate comparison. For comparing multiple datasets or more than two groups, ANOVA statistical tests were conducted in Prism. Results were expressed as means ± standard errors(SEM). Statistical significance was assigned at the level of *P* < 0.05.

## Results

### Characteristics of gene encoding *Sj* CP1412 mature peptide

**Fig. 1:**
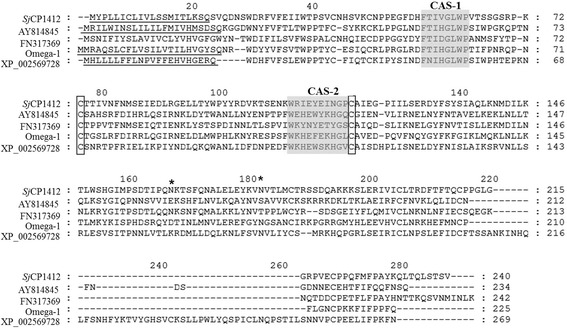
Homology comparisons of amino acid sequences of *Sj* CP1412,*Sj* AY814845, *Sj* FN317369, *Sj* FN 321900, *Sj* FN32424, *Sm* Omega-1 and *Sm* XP_002569728. Signal peptides are underlined, and the conserved functional domains (CAS-1 and CAS-2) of RNase T2 family are shaded in *gray*. Two conserved cysteine residues in the RNase T2 family are boxed. Potential N-glycosylation sites are labelled with *

A specific DNA fragment of ~666 bp was amplified from the plasmid pEU-GST-CP1412 by PCR and inserted directionally into the expression vector pET28a (+) at *Bam*H I and *Xho* I enzyme sites to construct the recombinant expression plasmid pET28a-r*Sj* CP1412 (see Additional file 3: Figure S3). The DNA sequence of the inserted fragment in r*Sj* pET28a-CP1412 was confirmed by restriction analysis of the recombinant plasmid of a single transformed colony and DNA sequencing. Bioinformatic analysis showed that the *Sj* CP1412 mature peptide is composed of 220 amino acids, with the predicted molecular weight of 25.2 kDa and isoelectric point of 6.15. The *Sj* CP1412 mature peptide also was predicted to contain two conserved functional domains (CAS-1 and CAS-2), a pair of conserved cysteine residues and two glycosylation sites of the ribonuclease T2 family [34, 35]. Analysis by amino acid sequence alignment showed that the similarities between *Sj* CP1412 protein and *Sm* Omega-1, *Sm* XP_002569728, *Sj* AY814845, *Sj* FN317369, *Sj* FN321900 and *Sj* FN324224 were 30.1, 24.2, 26.9, 35, 28.1 and 36.8%, respectively (Fig. 1). The low amino acid sequence homology among these proteins suggests that the *Sj* CP1412 protein is a novel member of the schistosome ribonuclease T2 family.

### Ribonuclease activity of r*Sj* CP1412 protein

**Fig. 2:**
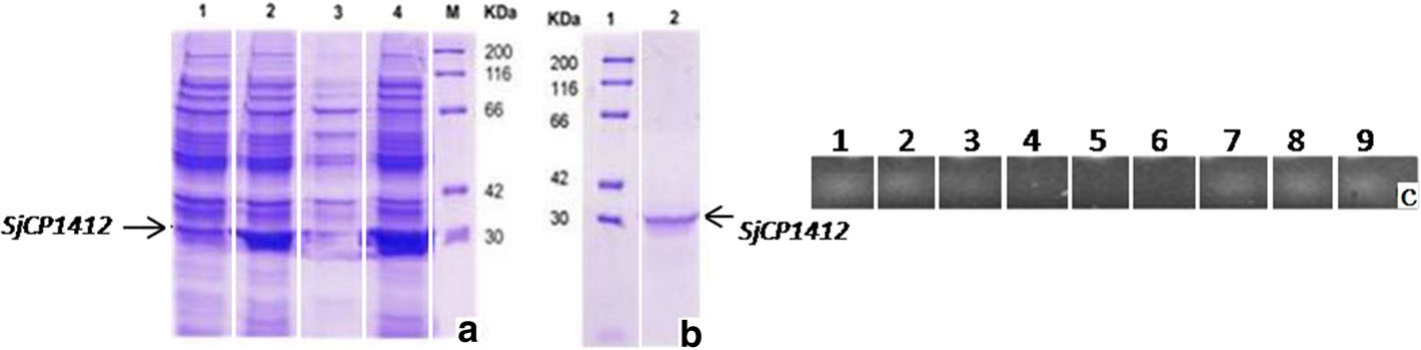
Analysis of expression and RNase activity of r*Sj* CP1412. **a** Lane 1: expression products of *E. coli* BL21 carrying plasmid pET28a-*Sj* CP1412 without IPTG induction; Lane 2: expression products of *E. coli* BL21 carrying plasmid pET28a-*Sj* CP1412 with IPTG induction; Lane 3: supernatant with expression products of *E. coli* BL21 carrying plasmid pET28a-*Sj* CP1412; Lane 4, precipitate of expression products of *E. coli* BL21 carrying plasmid *Sj*pET28a- CP1412; Lane M, standard protein molecular weight marker. **b** Lane 1: standard protein molecular weight marker; Lane 2: purified r*Sj* CP1412 protein. **c** Lane 1: yeast tRNA; Lane 2: RNA incubated inwater bath at 37 °C without *Sj* CP1412; Lane 3: products of RNA digested by 1.25 μg r*Sj* CP1412 protein; Lane 4: products of RNA digested by 2.5 μg r*Sj* CP1412 protein; Lane 5: products of RNA digested by 5 μg r*Sj* CP1412 protein; Lane 6: products of RNA digested by commercial RNase A; Lane 7: products of RNA digested by 1.25 μg rTg SAG1 protein; Lane 8: products of RNA digested by 2.5 μg rTg SAG1 protein; Lane 9: products of RNA digested by 5 μg rTg SAG1 protein

Bacterial transformants carrying the recombinant expression plasmid pET28a- r*Sj*CP1412 were cultured and induced by IPTG. The recombinant protein (r*Sj* CP1412) was found in the precipitates within inclusion bodies by analyzing expression products in the supernatant and precipitates of lysates by SDS-PAGE. The apparent molecular weight of r*Sj* CP1412 was ~30 kDa, similar to the predicted molecular weight (Fig. 2a). The r*Sj* CP1412 protein was prepared with high purity by affinity chromatography using a Ni-IDA affinity chromatography column in denaturing conditions. A portion of the r*Sj* CP1412 protein sample was renatured successfully into soluble proteins by gradient dialysis with different concentrations of urea in solution (Fig. 2b). The purified soluble r*Sj* CP1412 could mediate enzymolysis of yeast RNA, and its enzyme activity was positively correlated with the protein quantity (Fig. 2c). This result suggested that the purified r*Sj* CP1412 protein possessed RNase activity. However, compared with the activity of commercial RNase A, the activity of r*Sj* CP1412 in digesting RNA was weaker.

### Immunogenicity and expression levels of *Sj* CP1412 in different developmental stages of *S. japonicum*

**Fig. 3:**
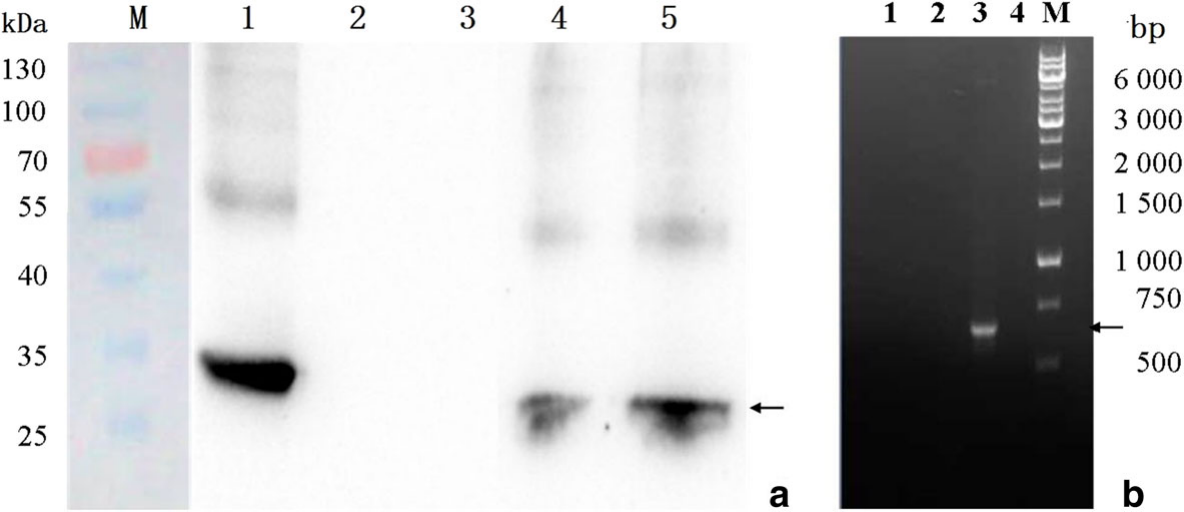
Expression analysis of *Sj* CP1412 in different developmental stages of *S. japonicum.* **a** Western blot analysis: Lane M, standard protein molecular weight marker; Lane 1: r*Sj* CP1412 recognized by anti r*Sj* CP1412 serum; Lane 2: soluble AWA of *S. japonicum* recognized by serum anti r*Sj* CP1412; Lane 3: SCA of *S. japonicum* recognized by anti r*Sj* CP1412 serum; Lane 4: SEA of *S. japonicum* recognized by anti r*Sj* CP1412 serum; Lane 5: ESA of *S. japonicum* egg recognized by anti r*Sj* CP1412 serum. **b** RT-PCR analysis: Lane 1: RT-PCR products of adult worm cDNA; Lane 2: RT-PCR products of cercaria cDNA; Lane 3: RT-PCR products of egg cDNA; Lane 4: negative control; Lane M: standard DNA molecular weight marker

Mice immunized with the r*Sj* CP1412 protein generated IgG antibody titers against r*Sj* CP1412 of more than 1: 200,000, suggesting that r*Sj* CP1412 is a strongly immunogenic protein. Western blotting analysis clearly showed protein bands in SEA and ESA with a molecular weight of ~25 kDa that could be recognized specifically by the sera against r*Sj* CP1412, while no protein band in AWA and SCA could be recognized by the same anti-serum (Fig. 3a). The intensity and size of the recognized protein band in ESA were higher and wider, respectively, than those of the recognized protein band in SEA, indicating that the quantity of *Sj* CP1412 in ESA was greater than that in SEA. PCR analysis using equivalent amounts of cDNA showed that a DNA band of the expected size (666 bp) for *Sj* CP1412 could only be amplified from the cDNA of eggs, but not from that of adult worms and cercariae of *S. japonicum* (Fig. 3b). These results indicated that the *Sj* CP1412 protein is only expressed in eggs and is an excreted protein.

### Effects of r*Sj* CP1412 on macrophage polarization

**Fig. 4:**
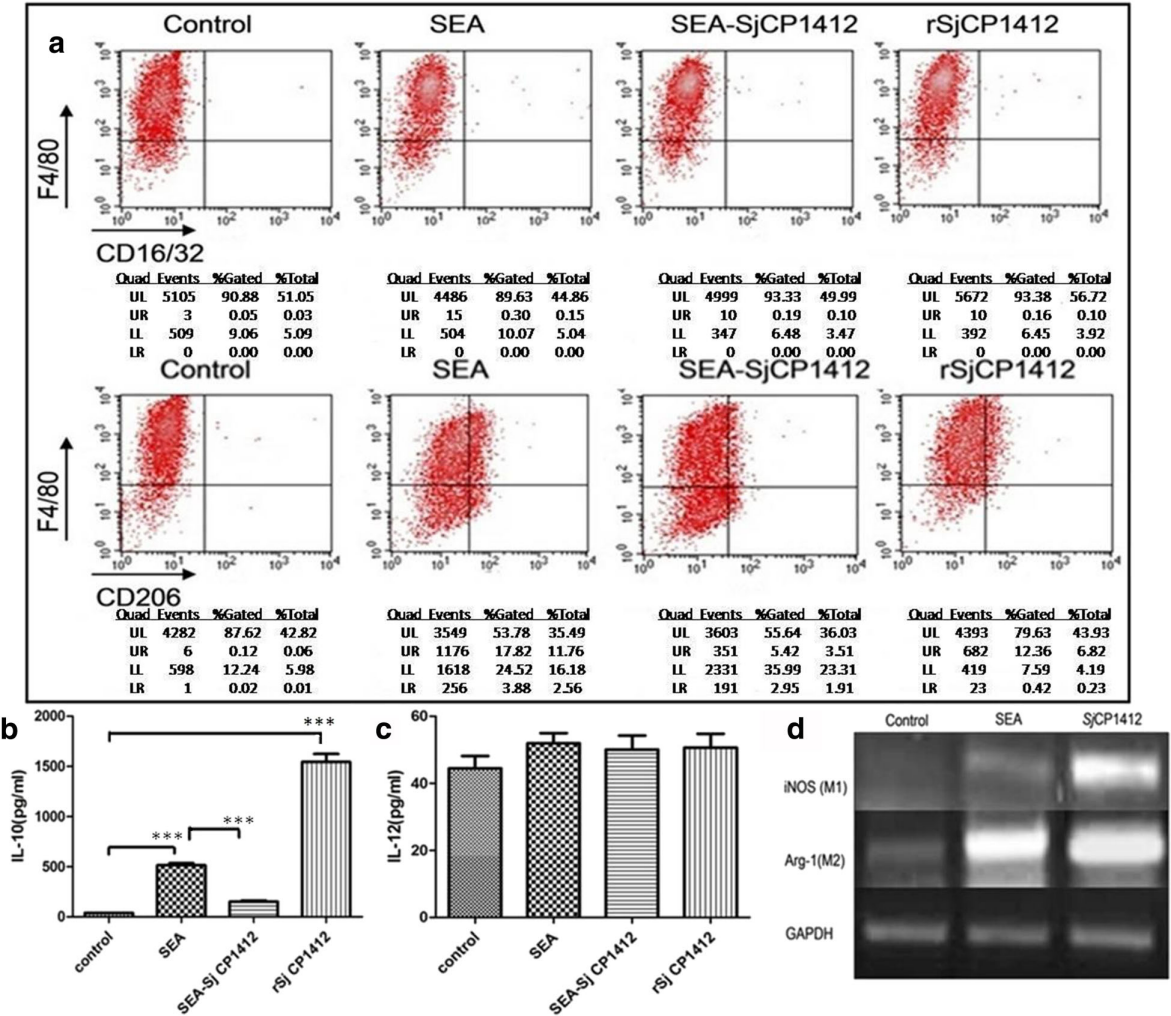
Effect of r*Sj* CP1412 on macrophage polarization. **a** Expression of CD16/32 and CD206 on surface of RAW264.7 cells stimulated by SEA, SEA-*Sj* CP1412 or and r*Sj* CP1412. **b** Levels of IL-10 in culture supernatant of RAW264.7 cells stimulated by SEA, SEA-*Sj* CP1412 or r*Sj* CP1412. **c** Levels of IL-12 in culture supernatant of RAW264.7 cells stimulated by SEA, SEA-*Sj* CP1412 or r*Sj* CP1412. **d** Expression of iNOS and Arg-1 mRNA of RAW264.7cells stimulated by SEA or r*Sj* CP1412. ***P < 0.001

FACS analysis of RAW264.7 cells stimulated with the purified r*Sj*CP1412and SEA in vitro showed that the expression of M2 type macrophage related surface marker CD206 of RAW264.7 cells was increased (*χ* ^2^ = 658.57, *P* < 0.0001 and *χ* ^2^ = 1232.84, *P* < 0.0001, respectively). Meanwhile, depletion of *Sj* CP1412 from SEA could downregulate the CD206 expression levels of RAW264.7 cells stimulated by SEA (*χ* ^2^ = 71.31, *P* < 0.0001; Fig. 4a). The M1 type macrophage related surface marker CD16/32 did not change significantly when RAW264.7 macrophage cells were stimulated by r*Sj* CP1412 (*χ* ^2^ = 0.222, *P* = 0.637) or SEA (*χ* ^2^ = 0.474, *P* = 0.491; Fig. 4a). The IL-10 level in the RAW264.7 cell culture supernatant stimulated by r*Sj* CP1412 or SEA was increased significantly (*t*_(4)_ = 24.23, *P* < 0.0001 and *t*_(4)_ = 71.68, *P* < 0.0001); however, the IL-10 level in the supernatant of RAW264.7 macrophages stimulated by *Sj* CP1412 depleted SEA was significantly decreased compared to that of RAW264.7 cells stimulated by SEA alone (*t*_(4)_ = 45.30, *P* < 0.0001; Fig. 4b). No obvious change of IL-12 level was observed in the stimulated RAW264.7 cells:*Sj* CP1412 (t_(4)_ = 1.004, *P* = 0.3721); SEA (*t*_(4)_ = 1.568, *P* = 0.1919) (Fig. 4c). The mRNA levels of iNOS and Arg-1 in the RAW264.7 macrophages stimulated by r*Sj* CP1412 were higher than those in the SEA group and PBS control, and the mRNA level of Arg-1 (M2-related) was obviously higher than that of iNOS (M1-related) (Fig. 4d). These results indicated that the *Sj* CP1412 protein has the ability to induce polarization of M2 type macrophages driving the host Th2 immune response.

### Effects of r*Sj* CP1412 on polarization of bone marrow DCs

**Fig. 5:**
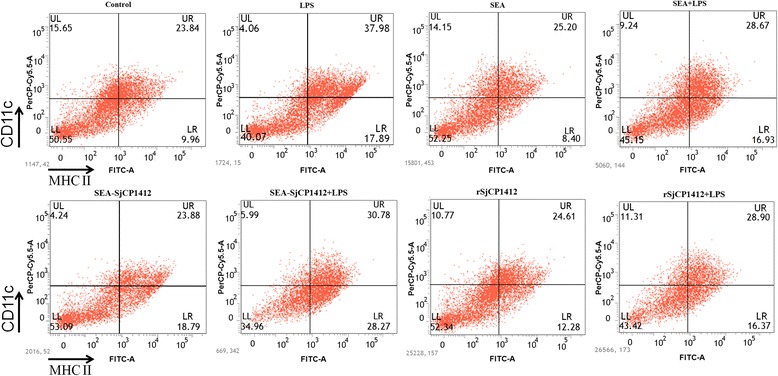
MHCII expression of DCs stimulated by LPS, SEA, SEA+ LPS, SEA-*Sj*CP1412, SEA-*Sj*CP1412 + LPS, r*Sj*CP1412, r*Sj*CP1412 + LPS, respectively, in vitro

**Fig. 6:**
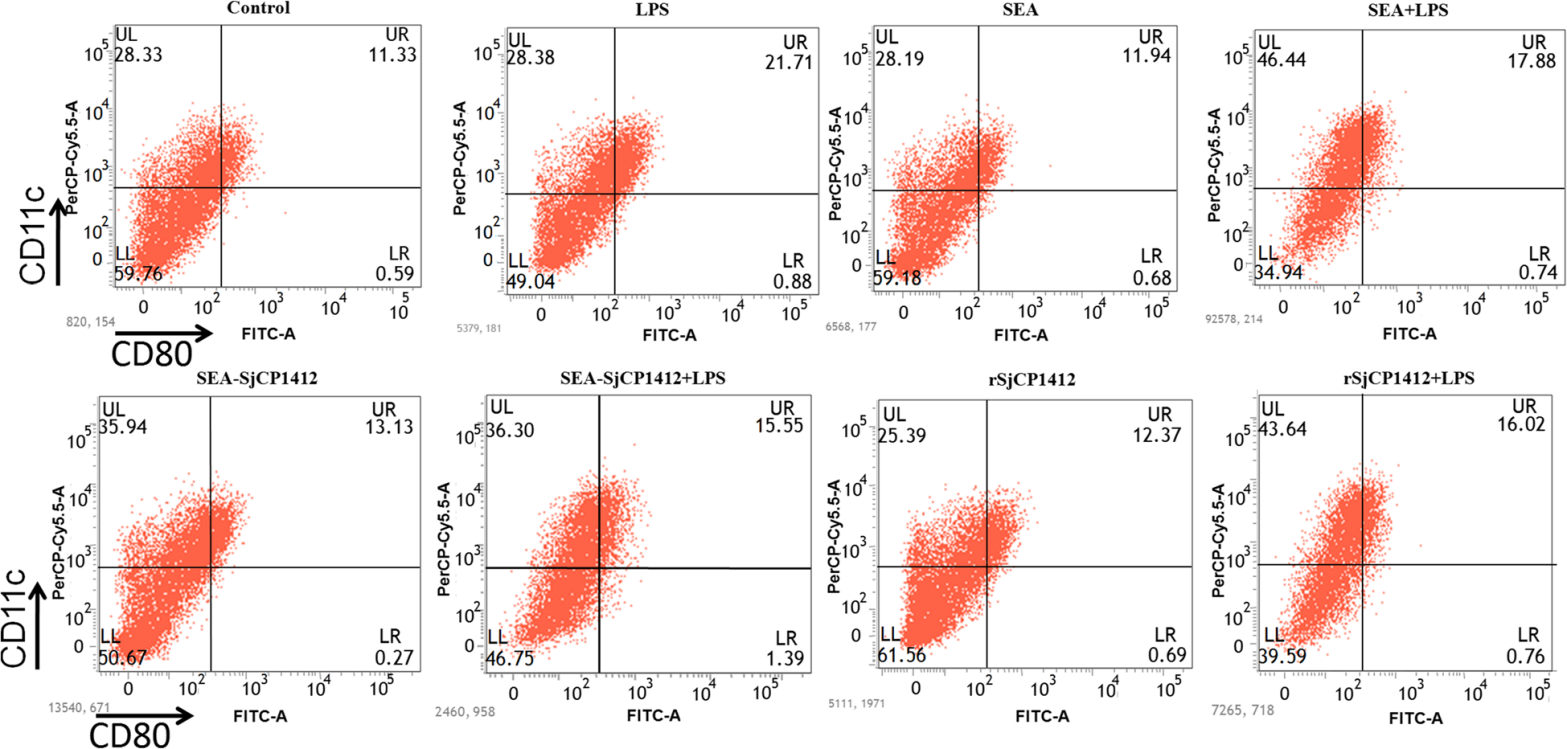
CD80 expression of DCs stimulated by LPS, SEA, SEA+ LPS, SEA-*Sj*CP1412, SEA-*Sj*CP1412 + LPS, r*Sj*CP1412, r*Sj*CP1412 + LPS, respectively, in vitro

**Fig. 7:**
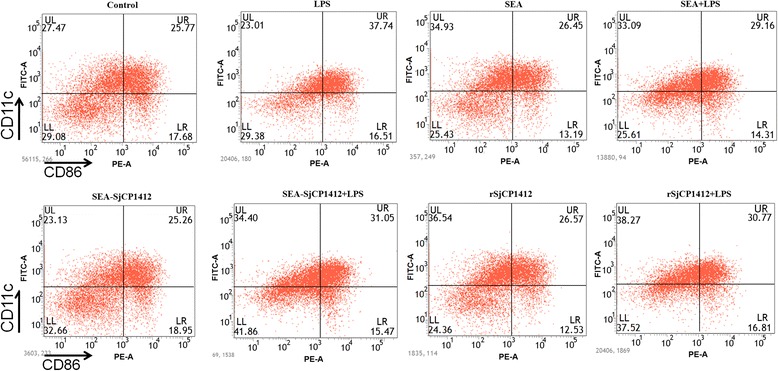
CD86 expression of DCs stimulated by LPS, SEA, SEA+ LPS, SEA-*Sj*CP1412, SEA-*Sj*CP1412 + LPS, r*Sj*CP1412, r*Sj*CP1412 + LPS, respectively, in vitro

DCs were stimulated with LPS, SEA, r*Sj* CP1412, *Sj* CP1412 depleted SEA alone, SEA plus LPS, r*Sj* CP1412 plus LPS or LPS plus *Sj* CP1412 depleted SEA in vitro for 24 h before expression levels of CD80, CD86 and MHCII on the DC surface were analyzed by FACS. The results showed that the LPS stimulation alone could significantly increase the MHCII (*χ* ^2^ = 525.125, *P* < 0.0001), CD80 (*χ* ^2^ = 390.461, *P* < 0.0001) and CD86 (*χ* ^2^ = 340.159, *P* < 0.0001) expression on the DC surface, but no significant effect of SEA (MHCII: *χ* ^2^ = 0.350, *P* = 0.067; CD80: *χ* ^2^ = 0.253, *P* = 0.133; CD86: *χ* ^2^ = 0.549, *P* = 0.459), r*Sj* CP1412 (MHCII: *χ* ^2^ = 1.537, *P* = 0.215; CD80: *χ* ^2^ = 1.632, *P* = 0.136; CD86: *χ* ^2^ = 1.196, *P* = 0.274) or *Sj* CP1412 depleted SEA stimulation alone on the expression of these markers was observed (MHCII: *χ* ^2^ = 0.380, *P* = 0.537; CD80: *χ* ^2^ = 0.651, *P* = 0.163; CD86: *χ* ^2^ = 0.884, *P* = 0.347). Expression levels of MHCII, CD80 and CD86 of DCs stimulated with SEA plus LPS (MHCII: *χ* ^2^ = 263.893, *P* < 0.0001; CD80: *χ* ^2^ = 46.338, *P* < 0.0001; CD86: *χ* ^2^ = 177.211, *P* < 0.0001), r*Sj* CP1412 plus LPS (MHCII: *χ* ^2^ = 218.553, *P* < 0.0001; CD80: *χ* ^2^ = 103.365, *P* < 0.0001; CD86: *χ* ^2^ = 126.934, *P* < 0.0001) or *Sj* CP1412 depleted SEA plus LPS (MHCII: *χ* ^2^ = 150.341, *P* < 0.0001; CD80: *χ* ^2^ = 124.707, *P* < 0.0001; CD86: *χ* ^2^ = 93.447, *P* < 0.0001) were significantly lower than those of DCs stimulated by LPS alone (Figs. 5, 6 and 7).

**Fig. 8:**
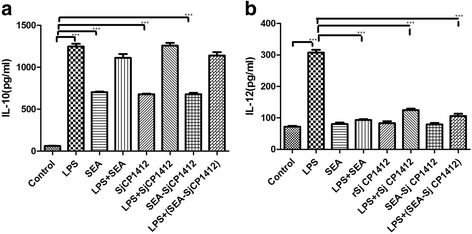
Effect of r*Sj* CP1412 on cytokine secretion of DCs. **a** IL-10 levels in supernatant of DCs stimulated by different antigens in vitro*.* **b** IL-12 levels in supernatant of DCs stimulated by different antigens in vitro*.* ***P < 0.001

Analysis of culture supernatants of stimulated DCs by ELISA showed that LPS stimulation alone could significantly increase secretion of IL-10 (*t*_(4)_ = 35.24, *P* < 0.0001) and IL-12 (*t*_(4)_ = 25.22, *P* < 0.0001). SEA (*t*_(4)_ = 102.1, *P* < 0.0001), r*Sj* CP1412 (*t*_(4)_ = 93.74, *P* < 0.0001) or *Sj* CP1412 depleted SEA (*t*_(4)_ = 38.88, *P* < 0.0001) stimulation alone also could significantly increase the levels of IL-10. The IL-10 level in the supernatant of cells jointly stimulated by LPS plus SEA (*t*_(4)_ = 2.383, *P* = 0.0757), LPS plus r*Sj* CP1412 (*t*_(4)_ = 0.2188, *P* = 0.8376) or LPS plus r*Sj* CP1412 depleted SEA (*t*_(4_ = 2.071, *P* = 0.1071) was similar to that of DCs stimulated by LPS alone, and no additive effect on IL-10 secretion was observed. r*Sj* CP1412 stimulation alone did not significantly affect the IL-12 secretion of DCs (*t*_(4)_ = 1.598, *P* = 0.185), which remained very low and nearly at the same level as that of the negative control; meanwhile, joint stimulation by SEA plus LPS (*t*_(4)_ = 23.07, *P* < 0.0001), r*Sj* CP1412 plus LPS (*t*_(4)_ = 18.04, *P* < 0.0001) and *Sj* CP1412 depleted SEA plus LPS (*t*_(4)_ = 17.31, *P* < 0.0001) actually inhibited the secretion of IL-12 from DCs compared with stimulation by LPS alone (Fig. 8a, b).

### Effects of r*Sj* CP1412 on polarization of host immune response and Treg cells

**Fig. 9:**
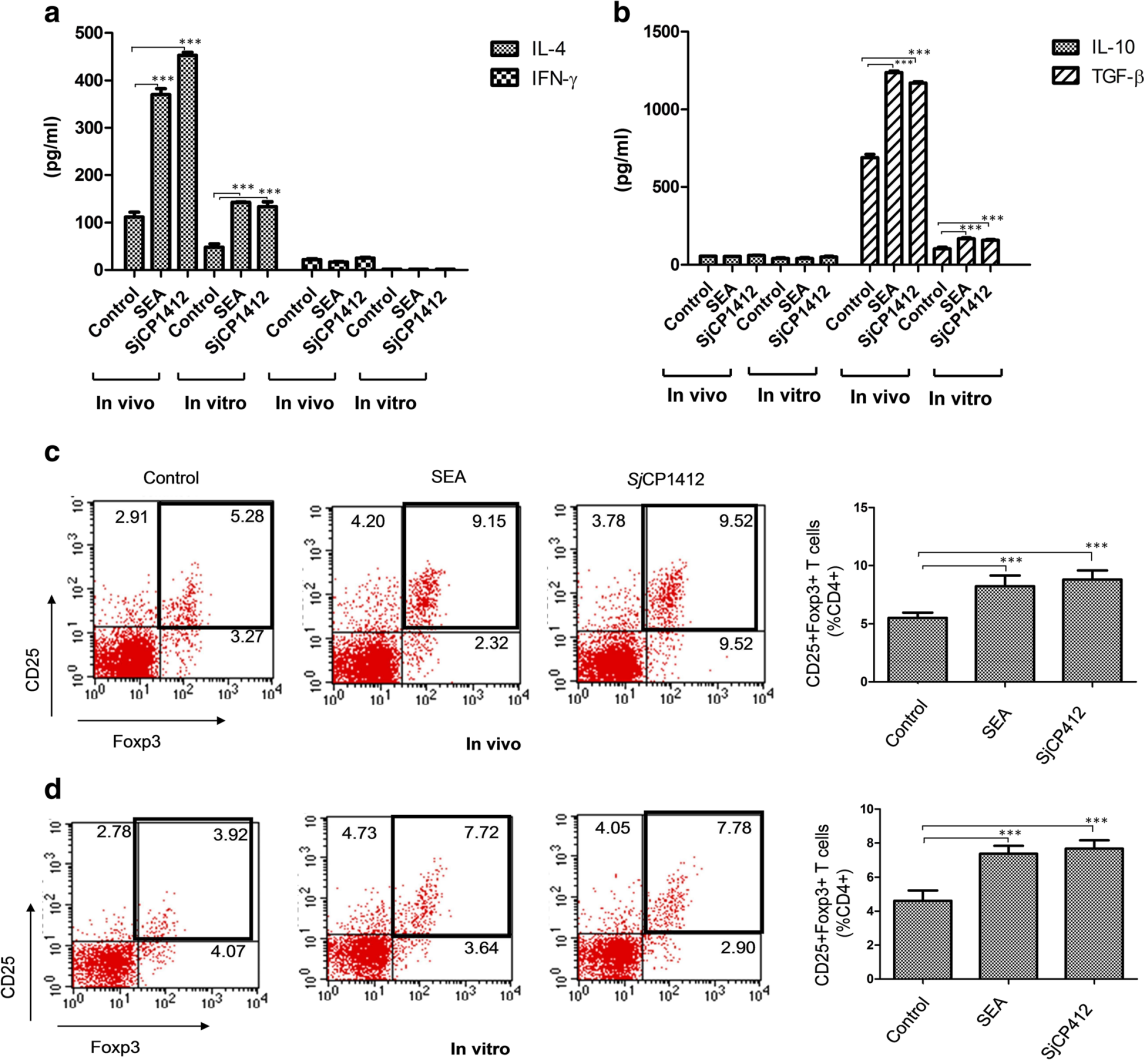
Polarization of immune response in mice immunized by r*Sj* CP1412. **a** Serum IL-4 and IFN-γ levelsin serum of mice immunized by r*Sj* CP1412 or in supernatant of splenic cells from immunized mice after stimulation by r*Sj* CP1412 in vitro. **b** IL-10 and TGF-β levelsin serum of mice immunized by r*Sj* CP1412 or in supernatant of splenic cells from immunized mice after stimulation by r*Sj* CP1412 in vitro. **c** Proportion of CD4 + CD25 + Foxp3+ T cells in splenic cells of mice immunized with r*Sj* CP1412. **d** Change in proportion of CD4 + CD25 + Foxp3+ T cells in splenic cells of healthy mice after being stimulated by r*Sj* CP1412 in vitro*.* ***P < 0.001

Compared with the control immunization group, the serum IL-4 (*Sj* CP1412: *t*_(4)_ = 69.63, *P* < 0.0001; SEA: *t*_(4)_ = 38.42, *P* < 0.0001) and TGF-β (*Sj* CP1412: *t*_(4)_ = 39.26, *P* < 0.0001; SEA: *t*_(4)_ = 97.20, *P* < 0.0001) levels of mice immunized with r*Sj* CP1412 and SEA increased significantly on day 7 after the third immunization, while no obvious changes in IFN-γ (*Sj* CP1412: *t*_(4)_ = 1.353, *P* = 0.2476; SEA: *t*_(4)_ = 0.1767, *P* = 0.1520) and IL-10 (*Sj* CP1412: *t*_(4)_ = 2.745, *P* = 0.0516; SEA: *t*_(4)_ = 0.3880, *P* = 0.7178) levels were observed. Meanwhile, cytokines in culture supernatants of control mouse spleen cells after 48 h of in vitro stimulation by r*Sj* CP1412 or SEA were detected by ELISA, and the results showed that IL-4 (*Sj* CP1412: *t*_(4)_ = 24.10, *P* < 0.0001; SEA: *t*_(4)_ = 32.08, *P* < 0.0001) and TGF-β (*Sj* CP1412: *t*_(4)_ = 33.22, *P* < 0.0001; SEA: *t*_(4)_ = 43.88, *P* < 0.0001) levels were significantly higher than those of the PBS control group. No obvious differences were observed in secretion levels of IFN-γ (*Sj* CP1412: *t*_(4)_ = 1.225, *P* = 0.2879; SEA: *t*_(4)_ = 0.7746, *P* = 0.4818) and IL-10 (*Sj* CP1412: *t*_(4)_ = 2.549, *P* = 0.0634; SEA: *t*_(4)_ = 0.1510, *P* = 0.8873) between r*Sj* CP1412, SEA and PBS control immunized groups (Fig. 9a, b).

FACS analysis showed that the proportions of CD4 + CD25 + Foxp3 + T cells in mouse spleen cells of *Sj* CP1412 and SEA immunized mice were increased to 9.52 and 9.15%, respectively, and that of PBS immunized mice was 5.28% (Fig. 9c). These results indicated that the r*Sj* CP1412 and SEA immunizations could significantly increase CD4 + CD25 + Foxp3 + T cells in mice (*χ* ^2^ = 624.31, *P* < 0.0001 and *χ* ^2^ = 864.15, *P* < 0.0001, respectively). The elevation in the number of CD4 + CD25 + Foxp3 + T cells by r*Sj* CP1412 immunization was similar to that by SEA immunization, with no statistically significant difference between the effects of these two antigens (*P* > 0.05).

To further verify the above experimental results, the single-cell suspensions of spleen lymphocytes from healthy mice were prepared and stimulated with r*Sj* CP1412 or SEA in vitro for 48 h. Percentages of Treg cells in stimulated spleen cells were determined by FACS, and the results showed that the ratio of CD4 + CD25 + Foxp3 + T cells in CD4+ T cells of the r*Sj* CP1412 and SEA stimulation groups rose to 7.78 and 7.72%, respectively, while that of the PBS control group was 3.92% (Fig. 9d). These results indicated that r*Sj* CP1412 (*χ* ^2^ = 228.11, *P* < 0.0001) and SEA (*χ* ^2^ = 436.68, *P* < 0.0001) could effectively convert the CD4 + T cells in the splenocytes into CD4 + CD25 + Foxp3 + T cells in vitro, and no significant difference in these effects between r*Sj* CP1412 and SEA was observed (*χ* ^2^ = 0.368, *P* = 0.867).

**Fig. 10:**
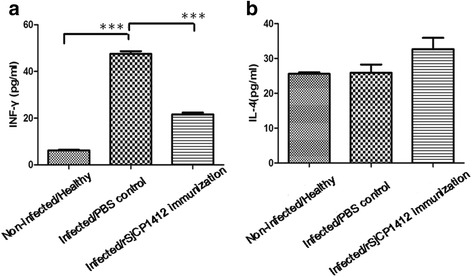
Cytokine levels in sera of immunized mice during early infection. **a** IFN-γ levels in sera of different mouse groups at day 21 post-infection. **b** IL-4 levels in sera of different mouse groups at day 21 post-infection. ***P < 0.001

**Fig. 11:**
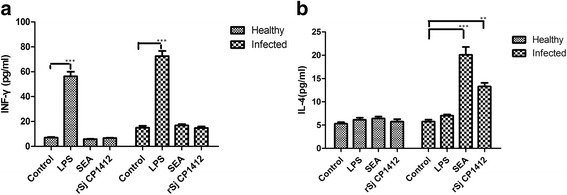
Cytokine levels in supernatant of spleen cells of immunized mice during early infection stimulated by different antigens. **a** IL-4 levels in supernatant of spleen cells of immunized mice and healthy mice. **b** IFN-γ levels in supernatant of spleen cells of immunized mice and healthy mice. **P < 0.01; ***P < 0.001

**Fig. 12:**
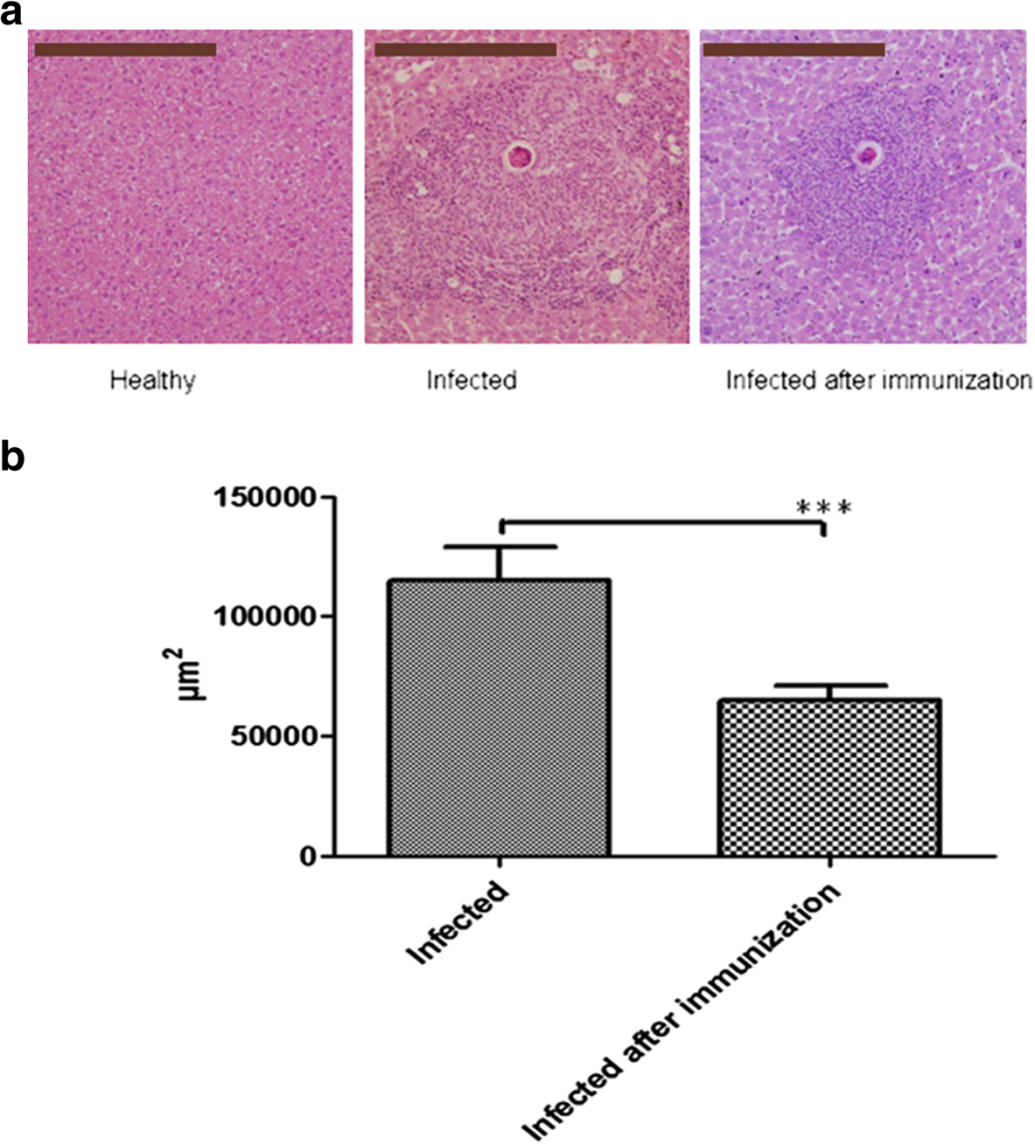
Effect of r*Sj* CP1412 immunization on egg granuloma pathology of mice. **a** Images of single egg granulomas in liver tissue sections of different mouse groups (original magnification × 100; *Scale-bars*: 200 μm). **b** Comparsion of the mean areas of single egg granulomas in mouse liver tissue sections of different groups. ***P < 0.001

In the early stage of schistosome infection of mice, the IFN-γ level in the sera was significantly higher than that in the r*Sj* CP1412 immunization/infection (*t*_(4)_ = 18.49, *P* < 0.0001) and healthy groups (*t*_(4)_ = 34.35, *P* < 0.0001) on day 21 post-infection (Fig. 10a). The IL-4 level in the sera of r*Sj* CP1412 immunization group rose slightly higher than that in the infection and healthy groups (Fig. 10b), but differences among the three groups were not significant (*t*_(4)_ = 1.692, *P* = 0.1659 and *t*_(4)_ = 2.171, *P* = 0.0957, respectively). The r*Sj* CP1412 could drive the proliferation of splenic cells in infected mice (*t*_(4)_ = 10.98, *P* < 0.001) (see Additional file 4: Figure S4) and enhance their IL-4 secretion after stimulation by r*Sj* CP1412 (*t*_(4)_ = 10.98, *P* < 0.001) or SEA (*t*_(4)_ = 8.542, *P* = 0.001) (Fig. 11a, b). The mean area of 22 single granulomas in livers of *Sj* CP1412 immunized mice at day 42 post-infection was smaller than that of the PBS group mice (*t*_(4)_ = 3.337, *P* = 0.0018) (Fig. 12a, b; see Additional file 5: Table S1). These results suggested that the Th2 type immune response was established at an early stage of schistosome infection in r*Sj* CP1412 protein immunized mice which could alleviate the egg granuloma pathology of schistosome infection.

### Effects of deactivated r*Sj* CP1412 on surface markers of RAW264.7 macrophages

**Fig. 13:**
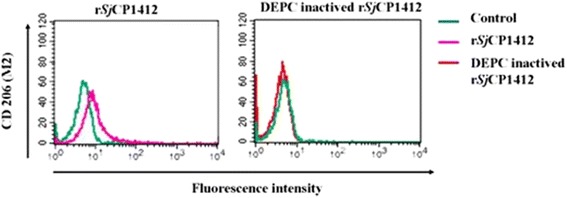
Effects of DEPC-inactivated r*Sj* CP1412 on phenotype of RAW264.7 cells

The r*Sj* CP1412 protein was treated with DEPC solution and then used to digest RNA samples. Analysis of the enzymolysis products by agarose gel electrophoresis confirmed that the DEPC-treated r*Sj* CP1412 protein completely lost its RNase activity (see Additional file 2: Figure S2). FACS analysis showed no significant change in the expression of surface molecule CD206 related to M2 type polarization of RAW264.7 macrophages which had been in vitro stimulated with DEPC-deactivated r*Sj* CP1412 (*χ* ^2^ = 823.56, *P* = 0.723), while the non-deactivated r*Sj* CP1412 could induce the macrophage expression of CD206 significantly (*χ* ^2^ = 565.31, *P* < 0.0001; Fig. 13); these results indicated that the immune regulatory function of *Sj* CP1412 is related to its RNase activity.

## Discussion

Many questions regarding schistosome pathobiology remain, including how the parasite manages to resist or evade the host immune system or why the host immune response shifts from a type Th1 response during the early infection to a type Th2 response at the late stage [12]. Antigens released from schistosomes, especially some of the excreted and secretory antigens of the schistosome egg, have been shown to regulate the host immune response actively [36]. The host antigen-presenting cells, such as DCs and macrophages, express Pathogen Recognition Receptors (PRR) to recognize and distinguish antigens molecules derived from different pathogens *via* their Pathogen Associated Molecular Patterns (PAMPs). Among them, the Toll-like Receptor (TLR) molecules have been explored intensively [37]. The carbohydrates derived from schistosome eggs have been shown to combine with TLR4 on DCs to prime the Th2 response [38, 39], and certain egg lipids can prime the Th2 response through TLR2 [40]. In addition, schistosome glycoproteins can also bind with DC-SIGN on the surface of DCs or bind with mannose receptors on the surface of macrophages for recognition and subsequent internalization, indicating that carbohydrate antigens can modulate DCs to drive the Th2 response through the C type lectin receptor (CLR) [16, 41].

In contrast to the classic activation phenomenon of microbial ligands *via* PRR activation of DC/macrophages, stimulation of these cells by parasitic antigens does not prime the classic activation but instead lead to immature DC cells and alternative activation of macrophages [42]. DCs exposed to carbohydrate molecules, such as SEA, ES-62 and LNFPIII, cannot promote p38 MAPK activation [43] but rather tend to induce ERK MAPK activation. Activation of the ERK pathway can inhibit the production of IL-12 and increase the secretion of IL-10 and TGF-β. IL-10 and TGF-β are important factors for promoting the transformation of Treg cells [44–46], and an increase in the number of Treg cells and enhancement of their function can effectively inhibit the host immune response. Le^x^ sugar molecules can also regulate LPS-induced signals by leukocyte specific protein-1 to increase the secretion of IL-10 and reduce the secretion of IL-12 in order to polarize the host immune response towards the Th2 type [47].

Antigens derived from schistosome eggs are potent inducers of the Th2 immune response, and many researchers have attempted to determine the specific functional molecules. Studies on *S. mansoni* have shown that molecules such as IPSE and Omega-1 in eggs can effectively induce the Th2 immune response in the host [29–31]. Induction of the Th2 immune response by the Omega-1 molecule was associated with its RNase activity, as the DEPC-deactivated Omega-1 could not carry out this function [30]. Omega-1 is an excreted protein molecule that can directly come into contact with the host immune system and has been proven to be a major immunomodulatory molecule in *S. mansoni* eggs [48]. In this study, the gene encoding the mature peptide of the *Sj* CP1412 protein was cloned, which had been identified by a SST method as an excreted protein of *S. japonicum* eggs. The mature peptide of the *Sj* CP1412 protein was prepared by the prokaryotic expression method (Fig. 2a, b), and bioinformatic analysis showed that it contains two conserved functional domains (CAS-1 and CAS-2) and two conserved cysteine residues of the ribonuclease T2 family, which are related to RNase activity (Fig. 1). Enzymatic analysis confirmed that the r*Sj* CP1412 protein could digest RNA, although its activity was weaker than that of commercial RNase (Fig. 2c). This lower potency may be attributed to the fact that the r*Sj* CP1412 protein was expressed in a prokaryotic system and may not have undergone all appropriate post-translational modifications or not folded correctly.

Analysis of amino acid homologies betweenthe *Sj* CP1412 protein and other members of the schistosome ribonuclease T2 family, such as *Sm* OMEGA-1, *Sm* RNase, *Sj* AY414845, *Sj* FN317369, *Sj* FN321900 and *Sj* FN324224, were lower than 40%. These results confirmed that the *Sj* CP1412 protein is a novel member of the ribonuclease T2 molecule family of schistosomes. To explore the immune regulatory function of *Sj* CP1412, mice were immunized with the r*Sj* CP1412 protein in this study and with SEA in parallel as a control. Both r*Sj* CP1412 and SEA were found to induce the Th2 polarization of the mouse immune response and raise cytokine levels of IL-4 and TGF-β related to the Th2 response (Fig. 9a, b). However, no significant change of cytokine levels of IL-12 and IFN-γ related to the Th1 response in sera of the immunized mice was found (Fig. 9a, b), and the number of Treg cells increased significantly in the spleen of immunized mice (Fig. 9c).

The in vitro stimulation experiments showed that the r*Sj* CP1412 protein could promote the differentiation of RAW264.7 macrophages toward the M2 type driving the Th2 immune response, increase the surface marker CD206 expression (Fig. 4a) and raise the IL-10 secretion (Fig. 4b) in stimulated macrophages. The expression level of Arg-1 closely associated with the M2 type of differentiation was clearly higher than that of iNOS-1 related to M1 differentiation in stimulated macrophages (Fig. 4d). Macrophages expressing high levels of Arg-1 can drive the Th2 polarization [49]. When DCs were stimulated by r*Sj* CP1412, they could not mature, with no significant change in the expression of the surface molecules MHCII, CD80 and CD86. However, stimulating DCs with SEA plus LPS, *Sj* CP1412 plus LPS, or *Sj* CP1412 depleted SEA plus LPS reduced the expression of the surface molecules of MHCII, CD80 and CD86 (Figs. 5, 6 and 7), which suggested that *Sj* CP1412 protein could inhibit the maturation of DCs induced by LPS. The IL-10 secretion of DCs stimulated by r*Sj* CP1412, *Sj* CP1412 depleted SEA or SEA alone was increased, but no additive effect on IL-10 secretion was found when DCs were stimulated by *Sj* CP1412 plus LPS, *Sj* CP1412 depleted SEA plus LPS, or SEA plus LPS (Fig. 8a). These results were consistent with reports of *S. mansoni* SEA not influencing the surface molecules related to the maturity of DCs, downregulating the expression of the surface co-stimulatory molecules and increasing the IL-10 secretion of DCs [15]. The above results show that the *Sj* CP1412 molecule can stimulate the major antigen presenting cells of DCs and macrophages and drive their differentiation toward the direction of facilitating the Th2 immune response. The level of IL-10 secretion in macrophages induced by r*Sj* CP1412 was significantly higher that induced with the same amount of SEA. Depleting *Sj* CP1412 from SEA could significantly impair its capacity to increase IL-10 secretion (Fig. 4b) and upregulate the expression of CD206 in RAW264.7 macrophages (Fig. 4a). The increase of Treg cells is a sign of Th2 type immune response polarization. Specifically, many research studies have demonstrated that the increase of Treg cells during schistosome infection is accompanied by Th2 polarization of the host immune response [50–52]. This study found that the abundance of CD4 + CD25 + Foxp3 + Treg cells increased significantly in the mouse spleen after immunization with r*Sj* CP1412 (Fig. 9c). In vitro stimulation tests confirmed that the r*Sj* CP1412 protein, like SEA, could promote the conversion of healthy mouse spleen cells into Treg cells in vitro (Fig. 9d) and increase the TGF-β secretion of stimulated cells (Fig. 9b). TGF-β has been shown to be a key cytokine of Treg cell differentiation [46]. Together, these results suggest that the *Sj* CP1412 protein has the ability to change ordinary CD4 + T cells of splenocytes into Treg cells. However, the question of whether pure CD4 + T cells can be translated into Treg cells by r*Sj* CP1412, or whether this translation requires other auxiliary factors (e.g. immune cells and/or cytokines), as well as the molecular basis and signaling pathways will need to be studied further.

As the r*Sj* CP1412 immunization of mice could inhibit inflammation in the early stage of schistosome infection, the serum IFN-γ level of the r*Sj* CP1412 immunization/infection group was significantly lower than that of the infection group (*P* < 0.05) (Fig. 10a), and the IL-4 secretion from mouse splenic cells increased. The mean area of single egg granulomas in the liver of r*Sj* CP1412 immunization/infection group was significantly smaller than that of the infection group (*P* < 0.05) (Fig. 12a, b; see Additional file 5: Table S1). These results demonstrated again that the Th2 type immune response induced by r*Sj* CP1412 immunization was established in the early stage of schistosome infection and could inhibit the development of liver egg granuloma inflammation in infected mice.

*Sm* Omega-1 has been shown to rely on its glycosylation sites to conjugate with MR receptors on the surface of antigen-presenting cells. After being internalized in the cell, the Omega-1 molecule degrades the mRNA on ribosomes and suppresses protein synthesis to induce the Th2 polarization of the host immune response [31]. As the r*Sj* CP1412 protein was expressed by prokaryotic cells in this study, it did not contain the glycosylation sites (see Additional file 6: Figure S5) and could not have entered cells in the same manner as *Sm* Omega-1 by binding with MR receptors. Therefore, *Sj* CP1412 molecules may gain entry into cells *via* another mechanism to exert its immune regulatory function. Our experimental results also showed that the DEPC-deactivated r*Sj* CP1412 protein lost the ability to induce the M2 type differentiation of macrophages (Fig. 13), indicating that the Th2 type polarization by *Sj* CP1412 relies on its ribonuclease activity. However, further investigation is necessary to determine how the *Sj* CP1412 protein molecules (including *Sm* Omega-1) can suppress protein synthesis in DCs and macrophages to drive the host immune response towards the Th2 direction and upregulate the number of Treg cells.

Western blotting and RT-PCR analyses showed that the *Sj* CP1412 protein is only expressed in eggs of *S. japonicum* and not in cercariae or adult worms. This expression pattern is similar to that of *Sm* Omega-1. Western blotting results also demonstrated that the abundance of *Sj* CP1412 in ESA was significantly greater than that of *Sj* CP1412 in SEA (Fig. 3a), which confirmed that *Sj* CP1412 is an excreted protein as predicted by the bioinformatic analysis. Feng et al. [53] reported that *Sj* AY814545 is expressed in adult worms only and not in the egg, unlike the expression pattern of *Sj* CP1412 and *Sm* Omega-1. As *Sj* AY814845 also contains conserved functional domains (CAS-1 and CAS-2) of the RNase T2 family, one may speculate whether it can regulate the host immune response similar to *Sm* Omega-1 and *Sj* CP1412. Using BLAST to search GenBank, three genes were found, including *Sj* FN317369, *Sj* FN321900 and *Sj* FN324224, which contain the conserved functional domains CAS-1 and CAS-2 and a pair of conserved cysteine residues of the RNase T2 family (Fig. 1). Issues regarding how these proteins are expressed in different developmental stages of *S. japonicum* and whether they participate in the regulation of host immune function are subject to further study.

## Conclusions

In summary, this study demonstrated that *Sj* CP1412 is an excreted protein expressed specifically in the egg and is a novel member of the RNase T2 family of *S. japonicum*. The r*Sj* CP1412 protein could induce the alternative activation of macrophages and immature DCs, increase IL-4, TGF-β and CD4 + CD25 + Foxp3 T cells of mice in vivo and drive the Th2 type polarization of the host immune response. The immune regulatory function of *Sj* CP1412 was associated with its ribonuclease activity, indicating that it may be an important immunomodulatory component in schistosome eggs. The above findings provide new evidence for further clarifying the role of *Sj* RNase T2 in the immune regulation of schistosome infection.

## Additional files

Additional file 1: Figure S1. Preparation of *Sj* CP1412 depleted SEA immune magnetic bead absorption method. Lane 1: *Sj* CP1412 depleted SEA could not be recognized by antibody IgG against r*Sj* CP1412, which indicated the *Sj* CP1412 in the SEA had been exhausted by immune magnetic bead absorption method. Lane 2: SEA (containing *Sj* CP1412) could be recognized by antibody IgG against r*Sj* CP1412. Lane 3: The recombinant *Sj* CP1412 was recognized by antibody IgG against r*Sj* CP1412. Lane 4: Prestained standard protein molecular weight marker. (TIF 51 kb)
Additional file 2: Figure S2. DEPC inactivated the RNase activity of r*Sj* CP1412 protein. Lane 1: RNA incubated at 37 °C water bath without *Sj*CP1412 (negative control); Lane 2: RNA digested by 5 μg DEPC inactivated r*Sj*CP1412 protein; Lane 3: RNA digested by 2.5 μg r*Sj*CP1412 protein; Lane 4: RNA Digested by 5 μg r*Sj*CP1412 protein (TIF 55 kb)
Additional file 3: Figure S3. Clone and recombinant plasmid construction of the gene encoding mature peptide of *Sj* CP1412. **a** Amplification of the gene encoding mature peptide of *Sj* CP1412. Lane 1: Molecular weight marker of standard DNA; Lane 2: PCR product of *Sj*CP1412. **b** Restriction analysis of recombinant pET28a-*Sj* CP1412 plasmid. Lane 1: Strandard molecular weight marker of DNA; Lane 2: Recombinant plasmid pET28a-*Sj*CP1412; Lane 3: Products of recombinant plasmid pET28a-*Sj*CP1412 digested by restriction enzymes BamHI/XhoI. (TIF 158 kb)
Additional file 4: Figure S4. The proliferation of infection mice spleen cells stimulated by different antigens (TIF 518 kb)
Additional file 5: Table S1. Data of single egg granuloma area of infection mice and infection after immunization. (XLS 19 kb)
Additional file 6: Figure S5. Glycosylation analysis of r*Sj* CP1412. *Note*: SEA could be recognized by biotin labeled LCA, which indicated that SEA containing the glycosylation site; r*Sj* CP1412 could not be recognized by biotin labeled LCA, which indicated that r*Sj* CP412 do not contain the glycosylation site. (TIF 78 kb)

## Abbreviations

APC: Antigen presenting cell
Arg: Arginine
AWA: Adult worm antigen
BCA: Bicinchoninic acid
BMDCs: Bone marrow-derived DCs
CD: Cluster of differentiation
cDNA: complementary DNA
CEF6: Serological activity of a cationic fraction six
CLR: C type lectin receptor
DCs: Dendritic cells
DEPC: diethypyrocarbonate
ELISA: Enzyme-linked immunosorbent assay
ERK: Extracellular regulated protein kinases
ESA: Excreted and secretory antigen
FACS: Fluorescence activated cell sorting
FITC: Fluorescein isothiiocyanate
Foxp3: Fork protein transcription factors
GAPDH: Glyceraldehyde-phosphate dehydrogenase
HE staining: Hematoxylin eosin staining
IFN-γ: Interferon gamma
IL: Interleukin
iNOS: induced nitric oxide synthase
IPTG: Isopropyl-β-D- thiogalactoside
LB medium: Luria-Bertani medium
LCA: Lens culinaris lectin
Le^x^: Lewis X
LPS: Lipopolysaccharide
MAPK: Mitogen-activated protein kinases
MHCI, II: Major histocompatibility complex I, II
MR: Mannose receptor
Mφ: Macrophage
NC membrane: Nitrocellulose membrane
PAGE: Polyacrylamide gel electrophoresis
PAMP: Pathogen-associated molecular patterns
PBS: Phosphate buffered saline
PE-Cy5: Phycoerythrin/cyanine dye 5
PRR: Pattern recognition receptor
rmGM-CSF: recombinant mouse granulocyte-macrophage colony-stimulating factor
RNase: Ribonuclease
RT-PCR: Reverse transcription polymerase chain reaction
SCA: Soluble cercariae antigen
SDS: Sodium dodecyl sulfate
SEA: Soluble egg antigen
*Sj*: *Schistosoma japonicum*
*Sm*: *Schistosoma mansoni*
SST: Signal sequence trapping
TGF-β: Transforming growth factor beta
Th: T helper cells
TLR: Toll like receptor
TMB: 3,3’,5,5’-tetramethylbenzidine
TNF-α: Tumor necrosis factor alpha
Treg: Regulatory T cell
