# Supplementary figures and images for: Characterization of Schistosoma japonicum CP1412 protein as a novel member of the ribonuclease T2 molecule family with immune regulatory function
Source: Parasit Vectors. 2017 Feb 17;10:89. doi: 10.1186/s13071-016-1962-y (PMC5316207; doi:10.1186/s13071-016-1962-y)

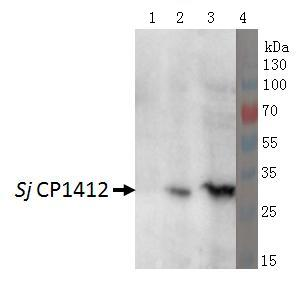

Supplement: Additional file 1: Figure S1. — Preparation of Sj CP1412 depleted SEA immune magnetic bead absorption method. Lane 1: Sj CP1412 depleted SEA could not be recognized by antibody IgG against rSj CP1412, which indicated the Sj CP1412 in the SEA had been exhausted by immune magnetic bead absorption method. Lane 2: SEA (containing Sj CP1412) could be recognized by antibody IgG against rSj CP1412. Lane 3: The recombinant Sj CP1412 was recognized by antibody IgG against rSj CP1412. Lane 4: Prestained standard protein molecular weight marker. (TIF 51 kb) [file 13071_2016_1962_MOESM1_ESM.tif]

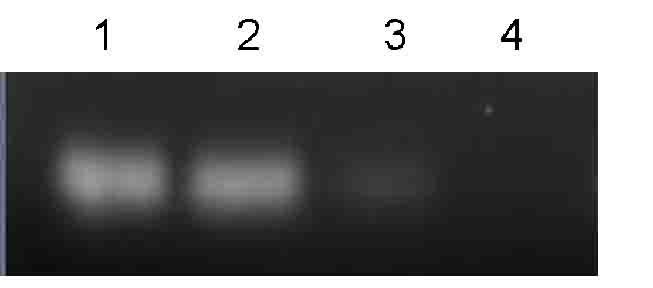

Supplement: Additional file 2: Figure S2. — DEPC inactivated the RNase activity of rSj CP1412 protein. Lane 1: RNA incubated at 37 °C water bath without SjCP1412 (negative control); Lane 2: RNA digested by 5 μg DEPC inactivated rSjCP1412 protein; Lane 3: RNA digested by 2.5 μg rSjCP1412 protein; Lane 4: RNA Digested by 5 μg rSjCP1412 protein (TIF 55 kb) [file 13071_2016_1962_MOESM2_ESM.tif]

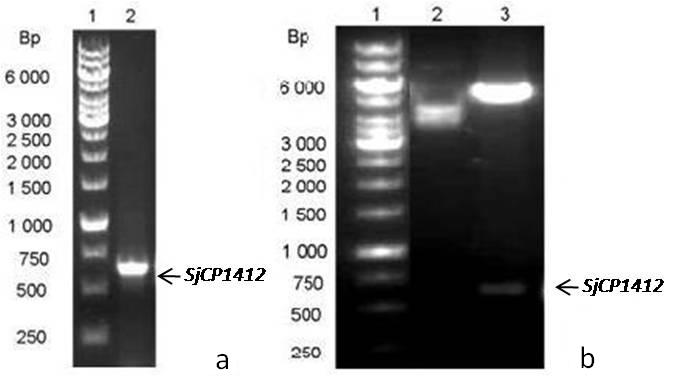

Supplement: Additional file 3: Figure S3. — Clone and recombinant plasmid construction of the gene encoding mature peptide of Sj CP1412. a Amplification of the gene encoding mature peptide of Sj CP1412. Lane 1: Molecular weight marker of standard DNA; Lane 2: PCR product of SjCP1412. b Restriction analysis of recombinant pET28a-Sj CP1412 plasmid. Lane 1: Strandard molecular weight marker of DNA; Lane 2: Recombinant plasmid pET28a-SjCP1412; Lane 3: Products of recombinant plasmid pET28a-SjCP1412 digested by restriction enzymes BamHI/XhoI. (TIF 158 kb) [file 13071_2016_1962_MOESM3_ESM.tif]

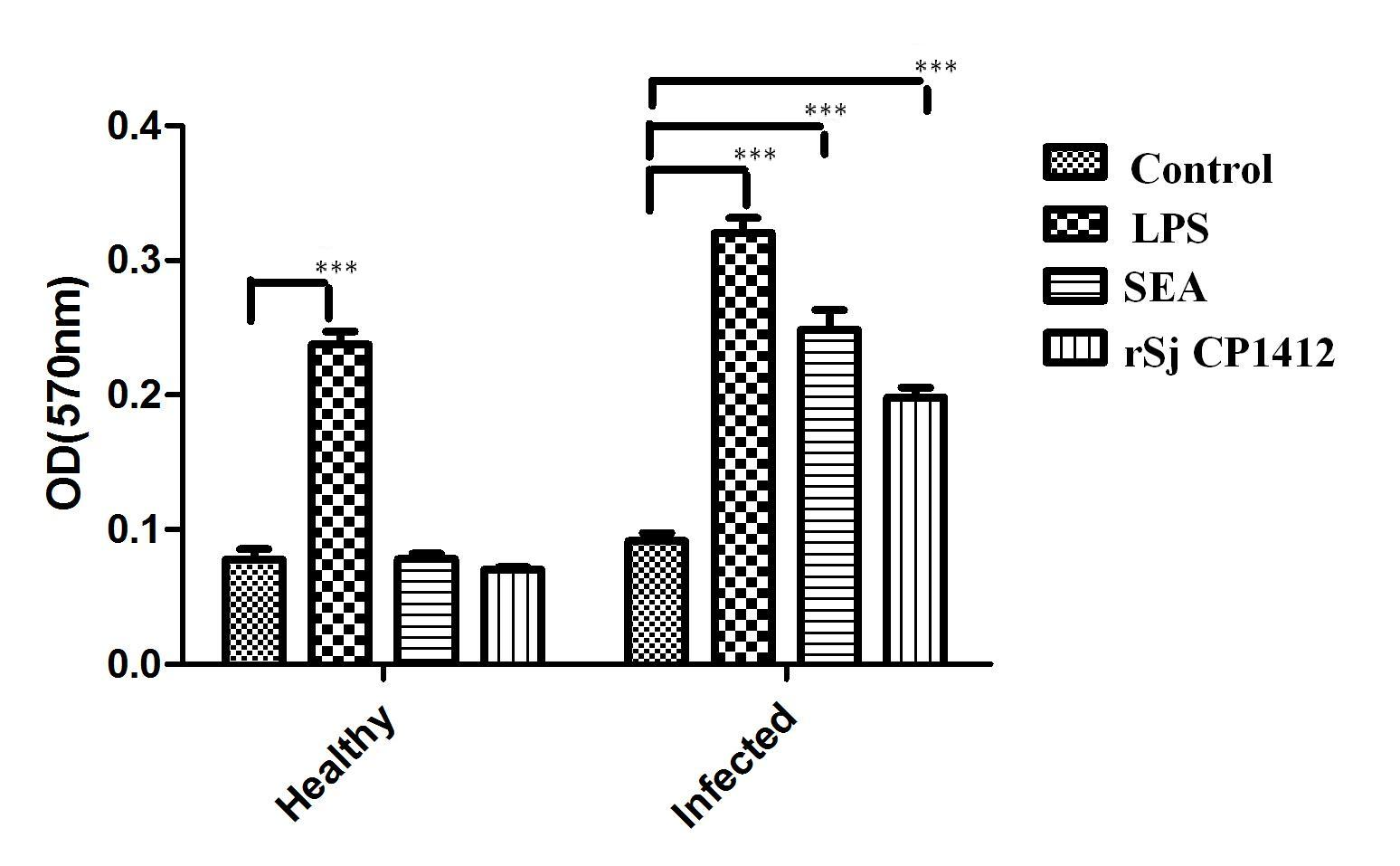

Supplement: Additional file 4: Figure S4. — The proliferation of infection mice spleen cells stimulated by different antigens (TIF 518 kb) [file 13071_2016_1962_MOESM4_ESM.tif]

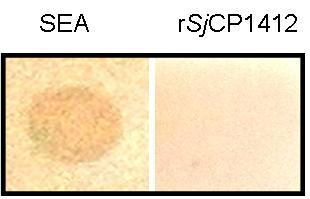

Supplement: Additional file 6: Figure S5. — Glycosylation analysis of rSj CP1412. Note: SEA could be recognized by biotin labeled LCA, which indicated that SEA containing the glycosylation site; rSj CP1412 could not be recognized by biotin labeled LCA, which indicated that rSj CP412 do not contain the glycosylation site. (TIF 78 kb) [file 13071_2016_1962_MOESM6_ESM.tif]
